# Auranofin‐Loaded PLGA Nanoparticles for Neuroprotection Against Aluminium‐Induced Alzheimer

**DOI:** 10.1002/alz70859_096807

**Published:** 2025-12-25

**Authors:** Rimpi Arora

**Affiliations:** ^1^ Christian Medical College and Hospital, Ludhiana, Punjab India

## Abstract

**Background:**

Currently, the treatment options for various CNS disorders, particularly neurodegenerative disorders, are greatly constrained. A significant obstacle in this pursuit is the blood‐brain barrier (BBB), a protective structure that hinders the route of numerous biochemical treatments into the brain. To overcome this problem nanoformulation‐based approaches are gaining interest, which increases the BBB penetrability of the compound.

The study aimed to assess the neuroprotective potential of auranofin‐loaded poly (lactic‐co‐glycolic acid) nanoparticles against aluminum chloride‐induced Alzheimer's Disease.

**Method:**

AF‐loaded PLGA nanoparticles were prepared and their entrapment efficiency, particle size distribution, surface charge, and morphology were characterized. In the in vivo study, AlCl_3_ (100 mg/kg, 21 days) was orally administered to rats, while, Auranofin and AF NPs (5, 10 mg/kg & 2.5 and 5 mg/kg) were administered orally for 2 weeks. After the treatment period, the rats were decapitated, and the hippocampus was collected for the estimated biochemical and neuroinflammatory markers.

**Result:**

The AF nanoparticles (AF NPs) were characterized, revealing an entrapment efficiency of 98%. The particle size was found to be 319.8±16.5 nm, with a surface charge of 27.5 and a polydispersity index of 0.438. In the in‐vivo study, the administration of both AF and AF NPs resulted in a significant reversal of cognitive deficits, biochemical alterations, and neuroinflammatory markers induced by AlCl_3_. These findings indicate that AF NPs exhibit a greater neuroprotective potential when compared to AF alone.

**Conclusion:**

The observed therapeutic benefits of the AF‐ PLGA nanoparticles can be attributed to modulation in particle size with antioxidant and anti‐inflammatory effects of Auranofin. Consequently, the outcome of the study reveals that the intervention holds gold nanoparticles as a potential candidate for the therapeutic modulation of Alzheimer's disease.

